# Plaque2.0—A High-Throughput Analysis Framework to Score Virus-Cell Transmission and Clonal Cell Expansion

**DOI:** 10.1371/journal.pone.0138760

**Published:** 2015-09-28

**Authors:** Artur Yakimovich, Vardan Andriasyan, Robert Witte, I-Hsuan Wang, Vibhu Prasad, Maarit Suomalainen, Urs F. Greber

**Affiliations:** Institute of Molecular Life Sciences, University of Zurich, Winterthurerstrasse 190, 8057 Zurich, Switzerland; Kliniken der Stadt Köln gGmbH, GERMANY

## Abstract

Classical plaque assay measures the propagation of infectious agents across a monolayer of cells. It is dependent on cell lysis, and limited by user-specific settings and low throughput. Here, we developed Plaque2.0, a broadly applicable, fluorescence microscopy-based high-throughput method to mine patho-biological clonal cell features. Plaque2.0 is an open source framework to extract information from chemically fixed cells by immuno-histochemistry or RNA *in situ* hybridization, or from live cells expressing GFP transgene. Multi-parametric measurements include infection density, intensity, area, shape or location information at single plaque or population levels. Plaque2.0 distinguishes lytic and non-lytic spread of a variety of DNA and RNA viruses, including vaccinia virus, adenovirus and rhinovirus, and can be used to visualize simultaneous plaque formation from co-infecting viruses. Plaque2.0 also analyzes clonal growth of cancer cells, which is relevant for cell migration and metastatic invasion studies. Plaque2.0 is suitable to quantitatively analyze virus infections, vector properties, or cancer cell phenotypes.

## Introduction

Plaque assay was originally developed for bacteriophages, and later adapted to mammalian viruses and eukaryotic cells [1, 2]. Plaques are clonal lesions or islets of infected cells formed by replicating viruses. Viruses form plaques by cell-to-cell or cell-free transmission, and elicit cytopathic effects [3, 4]. Yet, not all infections also lead to virus spreading and plaque formation, at least in part due to innate immunity [5, 6]. Plaques are used for clonal purification of agents from various etiologies, and for estimation of infectious titers. For example, virus titers are commonly expressed as plaque forming units (PFU).

Non-enveloped viruses often lyse infected cells, set free progeny and spread to neighboring cells, whereas enveloped viruses frequently spread by fusing infected with uninfected cells without appearance of extracellular virus, or by remaining tethered to the infected cell and lysing the infected cell after transmission [3, 4]. An example for a cell-to-cell spreading agent is vaccinia virus (VACV) from the *Poxviridae*, an enveloped brick shaped large DNA virus about 360 × 270 × 250 nm in size with a genome of 192 kbp [7–9]. Examples of viruses spreading via lytic cell-free route are human adenovirus (HAdV, genus mastadenovirus of the *Adenoviridae*) and human rhinovirus (HRV, genus enterovirus of the *Picornaviridae*). HAdV type C2 (HAdV-C2), is a non-enveloped virus with an icosahedral coat of about 90 nm in diameter, and a double-stranded DNA genome of ~36 kbp [10, 11]. Upon virus entry and replication HAdV-C2 progeny spreads lytic to neighboring airway epithelial cells [12–14], although non-lytic cell egress has also been reported under some circumstances [15]. HRV-A1A and HRV-A16 are small plus-sense single stranded RNA viruses with a non-enveloped icosahedral capsid of about 28–30 nm in diameter [16]. HRV replicates on Golgi-derived membranes in tight association with the ER, and can spread by lytic processes [17].

Many viruses use cell contact molecules as receptors, which suggests a spreading route through cell adhesion sites [18]. For example, herpes, measles or polio virus use nectins [19–22], hepatitis C virus uses claudin and occludin (for review, see [23]). HRV-A16 binds to ICAM-1 (intercellular adhesion molecule [1, 24]), HAdV-C2 and coxsackie virus use coxsackie virus adenovirus receptor (CAR), desmoglein or CD46, and adenovirus, rotavirus, herpes virus and a variety of picornaviruses take advantage of integrins (reviewed in [25, 26, 27]). For some of these cell adhesion receptors, it is known that they not just bind to the virus but also induce conformational changes in the bound virus and thereby prime the virus for further steps of entry, uncoating and infection [28–30].

Progeny viruses are formed and plaques appear after genome replication, transcription, translation, virus release from infected cells and infection of surrounding cells, frequently after cell killing. Interestingly, the shape of a plaque bears information about the transmission mode. Viruses, which spread exclusively by cell-cell mode form round plaques. If a virus spreads through the liquid cell-free medium, comet-shaped plaques emerge under the influence of convective micro-currents in the medium above the indicator cells [13]. Formation of the comet-shaped plaques can be prevented by gelling the medium, typically in conventional plaque assay. This reduces overlaps of lesion footprints and increases accuracy of titration, but masks information about the mode of viral spreading.

Recent advances in fluorescence microscopy and availability of transgenic fluorescent viruses together with immuno-staining of early or late viral proteins have allowed acquisition of multi-dimensional data from viral plaques [8, 13, 31]. In addition, viral plaques bear predictive information for *in vivo* virus spreading. For example, VACV forms circular plaques and efficiently spreads from cell-to-cell both in cell cultures and in organisms [32–34]. VACV spreading is mediated by the viral F11 protein, which inhibits Rho signaling, disrupts cell-cell contacts and enhances cell migration in the context of infection [35].

Here we present the Plaque2.0 software for automated analysis and quantification of virus spreading phenotypes. Plaque2.0 was developed for analyses of high-throughput midrange magnification fluorescence images. It yields multi-parametric measurements describing biological phenotypes. It extracts patho-biological information of clonal expansions of viral infection. Plaque2.0 scores and analyzes plaques from large enveloped DNA-viruses, such as the poxvirus vaccinia virus (VACV), non-enveloped DNA viruses, such as HAdV-C2, or non-enveloped small RNA viruses, such as HRV type A1A (HRV-A1A) and HRV-A16 from the picornavirus family. It also analyzes the formation of cancer cell colonies among normal cells in culture, which is a surrogate for metastatic cancer cell spreading in an organism.

## Materials and Methods

### Small molecular compounds and antibodies

Cytosine arabinoside (Ara-C, Sigma) [36] was dissolved in sterile double-destilled H_2_O (H_2_O) at 20 mM and stored at -20°C. Ara-C was added simultaneously with virus inoculum and kept in the medium throughout the experiment. Hoechst 33342 (Sigma) nuclear stain was dissolved in ddH_2_O at 2 mg/ml and stored at -20°C. It was used at 1:5000 dilution. Golgicide A (GCA, Calbiochem USA), which enhances HAdV infection [37], was dissolved in tissue culture grade sterile dimethyl sulfoxide (DMSO, Sigma) at 20 mM and stored at -20°C. GCA was used to pre-treat the cells 5 hours before infection. Cells were washed three times with phosphate buffered saline (PBS) and inoculated with virus. All experiments involving GCA were controlled with DMSO used to dissolve GCA. For scoring HRV infection, a polyclonal anti-HRV viral protein 1 (VP1) serum (VP1-89) was used to detect HRV-A1A and A16 infected cells at a dilution of 1:4000 (kindly provided by R. Valenta, Medical University of Vienna, [38]). Secondary goat-anti rabbit 488 antibody (Invitrogen) was used at 1:1000 dilution. Mouse monoclonal anti-ß-catenin (clone 14) was purchased from BD Biosciences (New Jersey, USA).

### Vaccinia viruses

Vaccinia virus strain Western Reserve (VACV-WR) and the strain International Health Department J (VACV-IHD-J) containing early / late (E/L) GFP transgene were provided by J. Mercer (University College London, UK) [39]. Mature virions (MVs) were purified from cytoplasmic lysates by pelleting through a 36% sucrose cushion for 90 min at 18,000 x g. The viral pellet was suspended in 10 mM Tris pH 9.0, and banded on a 25 to 40% sucrose gradient at 14,000 x g for 45 min. Following centrifugation, the viral band was collected by aspiration and concentrated by pelleting at 14,000 x g for 45 min. MVs were suspended in 1 mM Tris-HCl pH 9.0 and titered for plaque forming units (PFU) per ml as previously described [40]. The infectious particle to PFU ratio of VACV-WR and VACV-IHD-J virus on monkey kidney BSC40 cells was in the range of 50:1 and 80:1, respectively.

### Adenovirus

HAdV-C2_dE3B_GFP virus was produced as described [13]. Virus was grown in A549 cells and purified by double caesium chloride gradient centrifugation [41]. Aliquots were stored at -80°C. HAdV-C2_dE3B_GFP was found to be homogeneous by SDS-PAGE and negative-stain analyses by transmission electron microscopy.

### Rhinoviruses

HRVs used in the study were grown in HeLa cells as described [42, 43]. Cells were inoculated with a lysate from infected cells at 33.5° overnight. When CPE was visible in 80–90% of the cells, media was removed and cells harvested by scraping and pelleting. Cells were lysed by three freeze/thaw cycles followed by addition of 1% NP40 and homogenization with a Dounce homogenizer. Suspension was centrifuged at 2500 × *g* for 10 min and the supernatant transferred into a new tube. Free RNA was digested by addition of 150 μg RNase per 10 ml and incubation at 37°C for 30 min. Virus was purified on a CsCl gradient and extensively dialyzed against 140 mM NaCl, 25 mM Hepes, 5 mM MgCl_2_. Aliquots were stored at -80°C.

### Cell lines

Monkey kidney BSC40 cells were provided by J. Mercer (University College London, UK), HeLa cervical carcinoma cells strain Ohio were from L. Kaiser (University Hospital Geneva, Switzerland), and HeLa-ATCC from the American Type Culture Collection. Human lung carcinoma (HLC)-A549 cells (ATCC) were maintained in Dulbecco Modified Eagle Medium (DMEM; GIBCO-BRL) containing 10% fetal calf serum (FCS), non-essential amino acids (NEAA), and penicillin-streptomycin (GIBCO-BRL) at 37°C and 5% CO_2_ [44]. All cell cultures were maintained in a cell bank system and kept in low passages for all the experiments.

### Co-cultures of HeLa and WI-38 cells

HeLa cells expressing histone H2B (H2B)-mCherry allow specific visualization of their nuclei. These cells were co-cultured with non-labeled human lung fibroblast WI-38 cells in a 96-well imaging plate. A serial dilution of WI-38 was seeded first, resulting in varying confluency of WI-38 cells per well. HeLa cell suspension was added 24 h later. The amount of HeLa cells seeded was chosen to allow only few individual cells per well. Clonal growth of HeLa cells in wells with different confluency of WI-38 cells was observed by live cell imaging using automated epifluorescence microscopy, and samples were fixed in 4% PFA 5 days after seeding the HeLa cells. Cells were stained with Hoechst nuclear dye, immunostained for ß-catenin junction protein and imaged using confocal microscopy and automated epi-fluorescence microscopy.

### Conventional plaque assay

Plaque assays were performed with HeLa, BSC40 or A549 cells in DMEM cell-culture medium and 6-well tissue culture plates in liquid or semi-solid medium. VACV WR and IHD-J infections occurred at 37°C for 1 day, HAdV at 37°C for one week, and HRV at 33.5°C for 4 days in a 5% CO_2_ atmosphere. Cells were fixed and stained for 30 min with phosphate-buffered saline (PBS) solution containing 3 mg/ml crystal violet and 4% para-formaldehyde (PFA), added directly to the medium from a 16% stock solution. Plates were destained in H_2_O, dried and scanned using flatbed office scanner at 600 dots per inch (dpi).

Semi-solid medium was prepared using cell-culture-grade ultralow-melting-point agarose, type VII (Sigma, Fluka, Buchs, Switzerland). Agarose was dissolved in PBS, autoclaved and stored in aliquots at 3 mass percent. Before experiment, aliquots were heated to 90°C in a microwave oven, cooled down, controlled for liquid loss by gravimetry, adjusted with deionized H_2_O, diluted with warm cell growth medium to 0.6 mass percent agarose, overlaid onto cell monolayer and allowed to solidify at room temperature for 10 to 20 min.

### Comparison of conventional plaque assay and Plaque2.0

Conventional plaque assay was done in 6-well plates inoculated with 0.5 ml of inoculum medium containing VACV-WR, VACV-IHD-J, HAdV-C2_dE3B_GFP, HRV-A16 or HRV-A1A. In case of Plaque2.0 we used imaging compatible 96-well plates (Greiner Bio One) or 384-well plates (Matrix, Thermo Scientific) plates with the total inoculum volume of either 0.1 ml or 0.05 ml respectively. Plates were covered with either liquid culture medium or semi-solid medium, and fixed as described [13]. Plates were imaged using flatbed scanner (conventional plaque assay) or high-throughput microscopy (see below). Depending on the time post infection (pi) and virus, distinct lesions appeared in the middle of plaques, observable in the conventional plaque assay, and the Plaque2.0 assay by Hoechst nuclear staining, GFP transgene expression or immuno-staining. Additionally, in fluorescent plaques (foci) of all viruses we assayed, the most intense GFP or transgene-positive cell was usually situated in the center of the fluorescent focus.

### RNA fluorescence in situ hybridization (FISH) of HRV-A1A and HRV-A16 plaques

Reagents for RNA FISH staining were obtained from Affymetrix (Affymetrix UK Ltd.). Samples were fixed in formaldehyde (37% stock diluted 1:9 with PBS) for 30 min, washed in PBS, incubated with 0.05 ml “detergent solution QC” at 1:1 dilution with PBS for 3 min, rinsed three times with 0.1 ml of PBS. “Probe set diluents QF” was mixed 1:1 with “pre-hybridization buffer”, which was pre-mixed 1:1 with diethylpyrocarbonate (DEPC) treated H_2_O, heated to 40°C, mixed with target probe 1:100, vortexed briefly, and incubated with the cells at 50 μl/well 40°C for 3 h. Wells were washed three times with 100 μl wash buffer for 2 min each, and incubated with “pre-amplification mix” (prepared by diluting amplifier diluent stock 1:1 with “pre-hybridization buffer”) at 1:100 dilution in pre-warmed “amplifier diluent QF” at 50 μl/well) 40°C for 60 min.

Subsequently, wells were washed three times with 100 μl wash buffer for 2 min each. Working amplification mix was prepared by diluting amplifier diluent stock 1:1 with pre-hybridization buffer. Amplifier was added 1:100 in pre-warmed “amplifier diluents QF” (50 μl/well), vortexed briefly, added to wells and incubated at 40°C for 60 min, followed by three washes with 100 μl of wash buffer for 2 min. Finally, “label probe diluent” was mixed 1:1 with “pre-hybridization buffer” and used to dilute either type 1 label probe (HRV-A16, 550 nm) at 1:100, or type 4 (HRV-A1A, 488 nm) at 1:50. Mix was added to wells and incubated at 40°C for 60 min. Cells were washed three times with 0.1 ml wash buffer for 2 min and twice with 0.1 ml PBS at room temperature for 30 min, then stained with DAPI and imaged as described above.

### Midrange magnification high-throughput imaging

Multichannel microscopy images were recorded with an automated ImageXpress XL fluorescence microscope (IXM XL, Molecular Devices) using either a 4x Nikon S Fluor objective with a 0.2 numerical aperture (NA) or 10x Nikon Plan Fluor objectives with a 0.3 NA and equipped with multiple wavelength excitation/emission filters (Semrock). The full well was imaged at four sites (tiles). IXM XL is a high-throughput epi-fluorescence microscope equipped with automatic motorized stage, laser based autofocusing, 16-bit pco.edge sCMOS camera with resolution of 4.66 megapixel covering 3.5 x 3.5 mm at 4x, fluorescence filter cubes and a diode light source enabling imaging at 4 different wavelengths. A condenser yields transmission light (TL) images. Image acquisition was controlled using proprietary software based on Metamorph software—MetaXpress 3.0. Data management was based on MS SQL DBMS.

### Time-lapse multi-site, multi-channel microscopy

Time-lapse multisite, multichannel microscopy images were recorded with IXM XL microscope with a synthetic air-CO_2_ mixture of 95% and 5%, respectively, in a humidified environment at 37°C. All live imaging experiments were performed in 96-well black plates (Matrix; Thermo Fisher Scientific, Lausanne, Switzerland) and the entire wells were acquired in four sites by a motorized stage, allowing precise, high-speed selection of overlapping tiles.

### Confocal microscopy

Confocal fluorescence microscopy was conducted with a Leica SP5 confocal laser scanning microscope (Leica Microsystems, Germany) equipped with a 63x objective (oil immersion; NA 1.4), a UV laser (355 nm), an argon laser (488 nm), and a diode laser (561 nm). Z-stacks with optical sections spaced by 0.13 μm were acquired at 355, 488, and 561 nm excitation. Blind deconvolution and side projections of the z-stacks were performed by AutoQuant X3 software (Media Cybernetics, USA). The chromatic aberration was corrected based on bead images (4 μm TetraSpeck fluorescent microspheres, Life Technologies, USA) using Imaris software (Bitplane, Switzerland). Mouse monoclonal anti-beta-catenin (clone 14) was purchased from BD Biosciences (New Jersey, USA).

### Using the Plaque2.0 software

To make the software readily accessible, we have developed an end-user version with graphic user interface (GUI) based front-end. The GUI consists of four sections: input/output parameters, analysis flags, parameters and log sections. The software workflow is as follows. First, the user specifies the path to the input and output folders of the acquired images and the identifier pattern. Then user chooses from the analysis flags which steps to perform, and then specifies the required parameters in each of the selected steps. The active settings can be displayed by pressing “Test Settings” button in the corresponding analysis step tab. If imaging has been performed in a tiled acquisition manner (to obtain images of the full wells), stitching of the tiles is carried out. The number of sites that are stitched can be specified in the ‘Stitching’ panel. The program reads the images with the specified file masks for nuclei and virus signals from the “Raw Input Folder” and writes the output stitched images in the “Processing Folder”. This folder path is used for the latter analysis steps. Masking step defines the well mask, which can be defined manually, automatically or by loading a custom defined mask.

In addition, the software features various modules to enable analyses and define parameters. ‘Nuclei Tab’ defines parameters for nuclei detection. The user specifies the thresholding method choosing manual, global or local Otsu thresholding. “Artifact Threshold” determines the upper threshold for the images. This allows filtering out bright objects, such as dust particle artifacts. In case of Global Otsu thresholding, one can further tune the threshold using the “Minimum Threshold” and “Correction Factor” parameters. The former defines the lower boundary for the threshold detection. The latter is a user-defined arbitrary value by which the automatically detected threshold value can be multiplied to tune it up and down. In case of ‘Local Otsu method’ the program performs Otsu thresholding for parts of the images specified by the “Block Size” parameter. Minimum and maximum nuclei areas need to be empirically defined and are used to approximate the number of cells based on the thresholded images. To improve the quality of the thresholding the “Illumination correction” flag can be enabled. The magnitude of the correction can be controlled by modifying the “Gaussian Filter Sigma” parameter.

‘Virus Tab’ defines parameters for detection of plaque and infected cells. As the virus signal is usually more robust than the nuclear signal, manual thresholding is sufficient to distinguish foreground from background. This threshold is defined with the “Fixed Threshold” parameter. In order to filter single infected nuclei from plaque-like regions “Minimum Plaque Area” and “Connectivity” parameters need to be specified. “Connectivity” is the maximum distance between two neighboring pixels belonging to single plaque. “Minimal Plaque Area” is the minimum area of a foreground object that can be considered to be a plaque. When “Fine Detection” flag is enabled the program identifies local maxima inside detected plaque regions. “Gaussian filter size” and “Gaussian filter sigma” define the two dimensional Gaussian smoothing parameters. “Peak region” defines the maximum size of the region where the intensity maxima is detected. ‘Virus Segmentation’ quantifies infection signals based on a threshold which is selected by the user. Foreground pixels belonging to one plaque are then clustered or connected into a region based on the ‘*Connectivity’* parameter, which can be defined by the user. After all parameters have been set the user starts the analysis by pressing “Run” button on the input/output panel. All output of the software including errors is available in the log section. The results are exported to the specified “Result Output Folder” and saved as.csv and.mat files.

### Software license, set-up and dissemination

The Plaque2.0 software can be downloaded from http://plaque2.github.io/download.html. It has user-friendly GUI front-end and can be run on conventional workstations, or high-performance cluster computers through MATLAB. A detailed user manual is available at http://plaque2.github.io/help.html, together with installation and usage video instructions. Feature request and bug tracking is available at https://github.com/plaque2/matlab/issues. The source code can be found at https://github.com/plaque2/matlab. It was developed under the open source convention (GNU Public license V3, GPLv3, see http://www.gnu.org/licenses/) and is available on Github social coding platform together with documentation at http://plaque2.github.io. A compiled end-user version based on MATLAB compiler runtime (MCR, Mathworks) is available for all major platforms (Windows, Mac, Linux). MCR is free and the end-user does not require a MATLAB license to run the compiled Plaque2.0 software.

### Hardware requirements

Hardware employed for running Plaque2.0 is commercially available. Our microscopy was based on the IXM XL from Molecular Devices, and it can be compatible with other vendors of automated high-throughput microscopy solutions. Furthermore, any other fluorescence microscope or scanner capable of delivering images of similar quality as described here is compatible with our assay.

### Plaque2.0 in high-throughput mode

Conventional plaque assay in 6 well-plates uses solution volumes of about 0.5 ml. Plaque2.0 assays in 96 or 384 wells use volumes of 50–100 μl, and 30–50 μl respectively, using Liquidator (Mettler-Toledo). The time required to image a full 96-well or 384-well plate is about 15 min using high-throughput microscope and midrange magnification (4x). This comprised sequential imaging of 4 tiles, and two wavelengths with around 100 milliseconds exposure time per image. Importantly, the identification of microscopic plaques by Plaque2.0 shortens the time by more than 50% compared to conventional plaque assay.

### Z’-score analyses

The Z’-score was computed according to Eq (1)

$$Z\prime =1-\frac{\left(3\sigma_{C+}+3\sigma_{C-}\right)}{\left|\mu_{C+}-\mu_{C-}\right|}$$ (1)

where *σ*_*C+*_—is the standard deviation of the positive control, *σ*_*C-*_—standard deviation of the negative control, *μ*_*C+*_—mean of the positive control, *μ*_*C-*_ *—*mean of the negative control. Z’-score between 1 and 0.5 is well suited for HTS experiments [45].

### Software implementation, image processing and analysis pipeline

Plaque2.0 analyzes fluorescence intensity and distinguishes even partly overlapping plaques, as well as fluorescent markers for additional readouts, e.g. total count of cells. The software is written and compiled in MATLAB (Mathworks) using image processing toolbox. It consists of four different modules: stitching, masking, monolayer and plaque. The stitching module merges individual raw tiles images into a single image. The masking module creates an overlay mask over the stitched image. Monolayer module can be used for quantification of nuclear signals based on foreground pixels segmentation. The foreground pixels detection in nuclear images is done using Otsu thresholding method [46, 47]. Uneven illumination in images is corrected by subtracting the Gaussian convolution of the original raw image using “rolling ball” algorithm [48]. To circumvent limitations of midrange magnification and high confluency of the cell monolayer the nuclear signals and the virus signals are processed independently with respect to object/foreground detection, and are then cross-correlated to refine the image information. The calculated number of total cells is based on the number of foreground pixels defined by nuclear staining divided by the average nuclear size. The total number of infected cells is calculated similarly, that is by estimating the number of cells of interest based on the infection signal.

### Identification of plaques

Plaques (or foci) of fluorescent groups of cells are identified by global fixed thresholding interactively fine tuned by the user. For details, see user manual and help video at http://plaque2.github.io/. Each plaque contains an intensity maximum, which represents the founder cell, which was first infected. This defines the baseline of the ‘Coarse’ and ‘Fine’ detection algorithm utilized by the software, and allows determining an accurate count of plaques, even at their high density and partial overlapping. Specifically, the following procedure is used for plaque identification. First, based on the thresholded black and white images the maximum distance between neighboring nonzero pixels is determined. This procedure termed ‘Coarse Detection’ is performed by default. Next, all the elements from each non-zero pixel within the neighbor distance are connected to form the complete objects. Then smaller objects are filtered out based on user defined size constrains. Local fluorescence maxima within each plaque region are determined by smoothing the intensity profile of the detected plaque region and then identifying the peak fluorescence region (details are described in the online manual under http://plaque2.github.io/help.html). This feature is implemented in the software as ‘Fine Detection’, and corresponding settings can be found in the user interface. Hence, the cells positive for viral fluorescence that were directly connected and had a local maximum were identified as a plaque object.

### Features of plaques

Particular features of plaques can be extracted by treating plaques as objects. We have implemented algorithms to determine the plaque geometry, area, cell counts, the mean, maximum as well as total intensity of the infection signal. Such features are uncovered by taking the viral fluorescent micrographs and optionally the micrographs with nuclear stains of the cell monolayer as inputs. These features include the centroid coordinates, the bounding box within a fitted rectangular area, upper right corner coordinates and box dimensions, length of major and minor axes and convex area of the fitted ellipsoid. Further downstream in the pipeline, individual plaque objects (or overlapping plaque regions) resulting from this segmentation are characterized for object area, centroid coordinates of the fitted rectangle, upper right corner coordinates and rectangle dimensions, major and minor axis length of the fitted ellipsoid, convex area of the fitted ellipsoid, number of nuclei in the plaque, as well as mean, maximum and total intensity of the virus signal.

### Plaque co-localization measurement using object-based readouts

To determine the overlap between plaques detected by different markers from different fluorescence channels we used the location of the plaque centroids. For each centroid we computed Euclidean distances to the nearest neighbor plaque centroid from the second channel. In case of a perfect co-localization (e.g. in control case where arbitrary first and second channels were identical) all nearest neighbor distances should be very small or equal to zero. In case of no co-localization (random proximity) only few distances are expected to be close to zero. Two internal controls were introduced, self co-localization and random co-localization. For the former distances to the nearest neighbor were used when both channels were the same. For the latter, co-localization in case of two signals from two different wells was used, where nearest neighbor distance are expected to be purely coincidental and approach random.

### Eccentricity measurement

Eccentricity of an elliptical shape is defined as the fraction of the distance between the two focal points of the ellipse divided by the length of the major axis. Eccentricity of a circle (with one focal point) is zero. Eccentricity of a plaque or clone of cells is a standard readout provided by the software. It is useful for measuring how elongated the shape of the plaque is. For example in comet shaped plaques, which are characteristic for viruses that spread to neighboring cells by passive mass transfer.

### Crowdedness measurement

Among co-cultured WI-38 cells, the fraction of HeLa cells expressing H2B-mCherry, which were obtained from D. Gerlich [49] is referred to as crowdedness within an area of interest.

## Results

### Fluorescence microscopy extends conventional plaque assay

**Fig 1:**
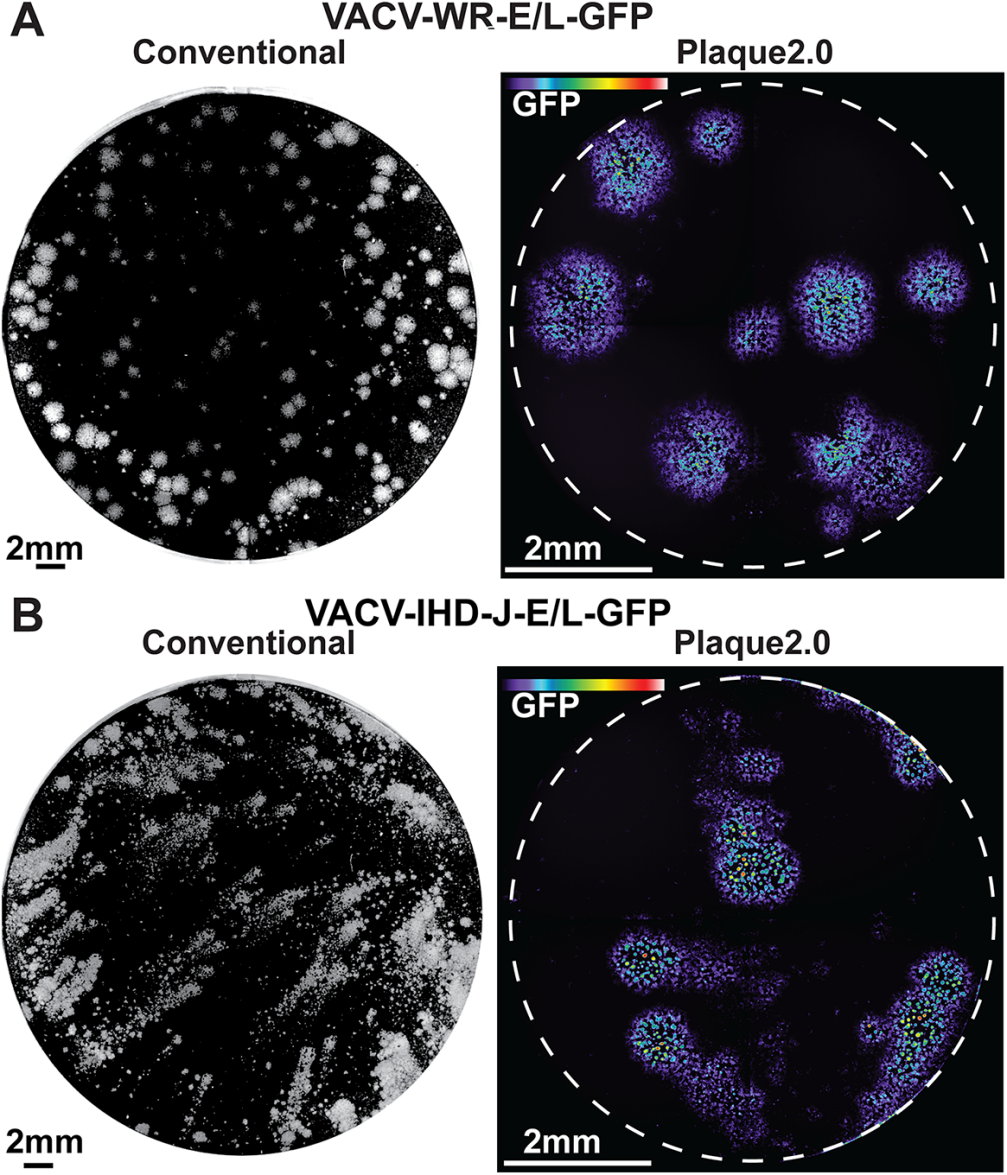
Conventional plaque assay and Plaque2.0. Comparison of conventional plaque assay and Plaque2.0 analyses of VACV-WR-E/L-GFP (A), or VACV-IHD-E/L-GFP (B) infections of BSC40 cells in liquid medium. Conventional plaque assays were carried out in 6-well plates with a total volume of 0.5 ml per well. Plaques appeared as white round or smeary comet-like objects after 2 days, as indicated by crystal violet staining of cells in the monolayer. Plaque2.0 analyses were carried out in 96-well plates using epi-fluorescence microscopy at 4x magnification. Four images were stitched to yield an image of the entire well. Color-coded GFP intensity is scaled from 0 (violet) to 6000 gray scale values (pseudo-colored in violet to deep red).

We explored if fluorescence imaging was suitable to measure viral plaque morphologies. For this we used two strains of VACV, the Western Reserve (WR) strain producing circular plaques, and International Health Department J (IHD-J) producing comet-shaped plaques [32, 34]. VACV-WR-E/L-GFP or VACV-IHD-J-E/L-GFP expressing GFP from early-late promoter was inoculated onto monkey kidney BSC40 cell monolayers in 96-well imaging plates, or 6-well plates for conventional plaque assay. Both plates were fixed 48 h pi. In both conventional and Plaque2.0 assays the IHD-J strain produced comet-shaped fluorescent foci, so called fluorescent plaques (in short ‘plaques’), while the WR strain produced circular plaques (Fig 1). VACV occurs in two infectious particles, the single membrane intracellular mature virions (IMVs), and double membrane virions referred to as cell-associated extracellular enveloped virions (CEVs) or extracellular enveloped virions (EEVs) [9, 50]. The former are predominantly cell-bound and the latter free in the extracellular medium. The comet-plaque forming IHD-J is known to produce significantly more EEVs than the WR strain [32]. This feature was confirmed by titrating infectious virus from the medium of WR and IHD-J infected cells (Figs A-C in S1 Fig). The results are consistent with the notion that comet-shaped plaques arise from extracellular viruses and move between cells by convection forces [13].

### Plaque2.0 software scores phenotypic features of vaccinia virus plaques

**Fig 2:**
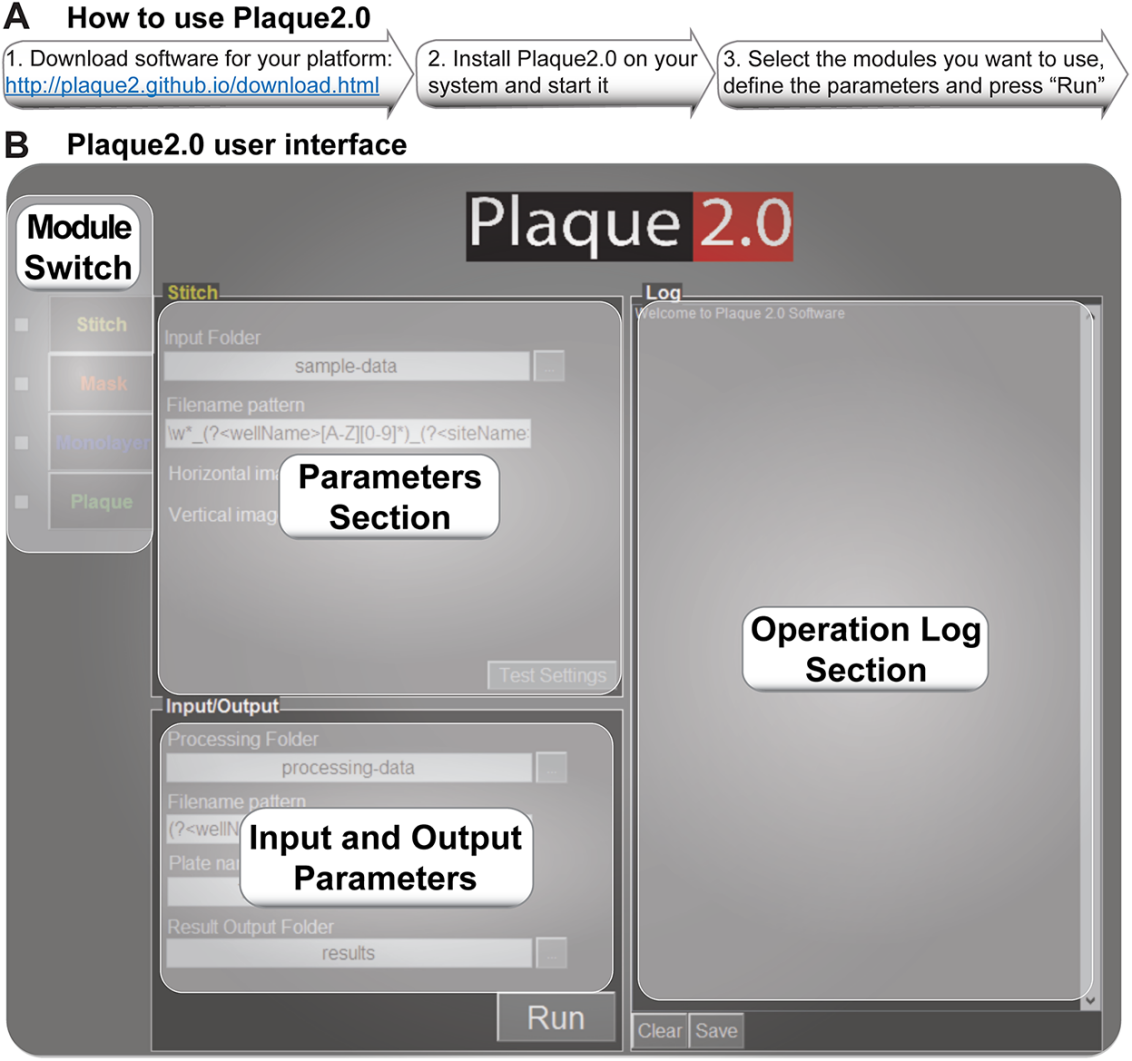
How to use Plaque2.0 software. (A) Downloading and using the Plaque2.0 software in three easy steps. (B) A graphical user interface (GUI) navigates through the “Operation Log” section with text output for all operations performed by the software, including potential errors. “Parameters Section” allows to specify user-defined values of the input parameters for all the software modules. “Analysis Type Switch” allows switching between parameters for individual modules, or activating and deactivating them.

Close inspection of the IHD-J fluorescent comet plaques revealed multiple foci of high signal intensity, akin to satellite plaques (Fig 1). To quantitatively assess plaque features we developed a modular open source MATLAB based software framework. Installation instructions for Windows are available in a video on ‘youtube’ (https://youtu.be/DAZ1OPXrW9A). The user can download the standalone software for a platform of choice (Windows, Mac, Linux) from http://plaque2.github.io/download.html, set the parameters for analyzing fluorescent plaque images, and run the analyses (Fig 2A). A graphical user interface (GUI) eases the use of the software (Fig 2B). A typical image analysis procedure involves stitching the tiled images to a full well image using ‘stitch’ module. Then the region of interest is selected using the ‘mask’ module to exclude image artifacts, and plaques are scored using ‘plaque’ module. Detailed procedures are available at http://plaque2.github.io/help.html, and a help video is provided at http://plaque2.github.io/.

**Fig 3:**
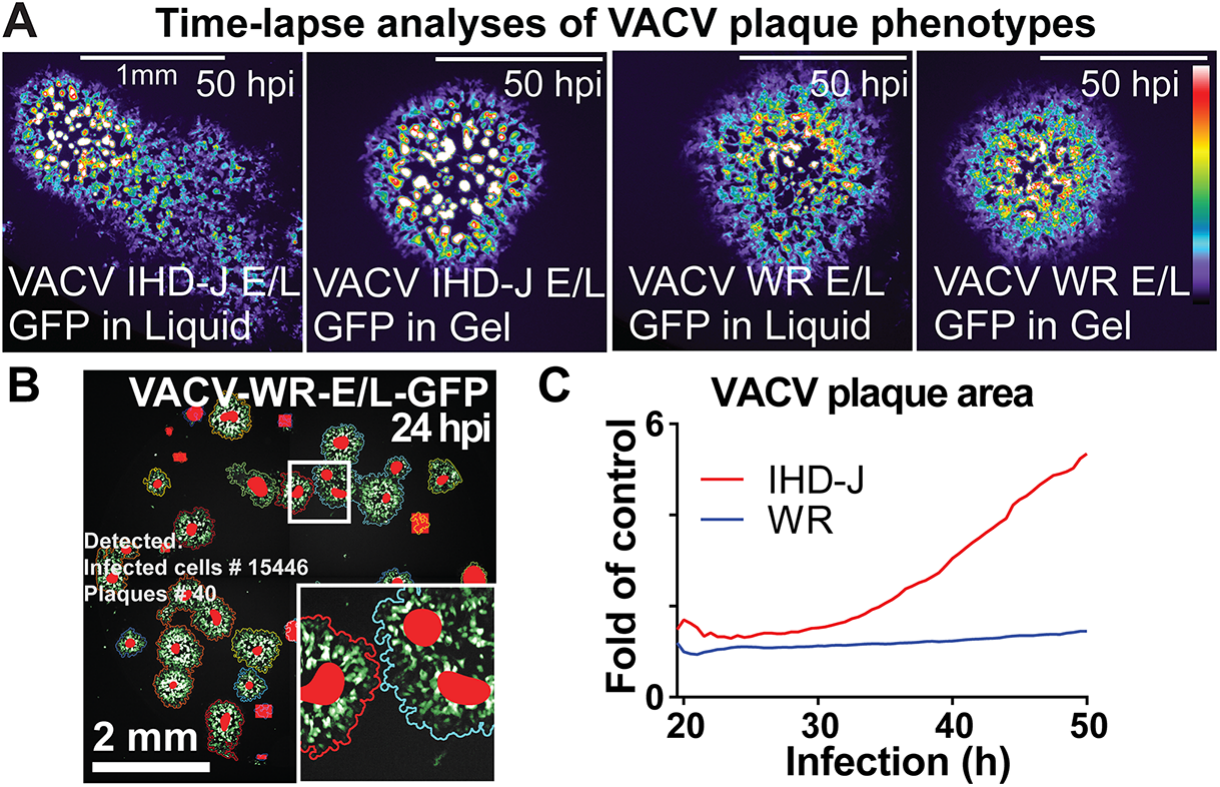
Time-lapse analyses of plaque phenotypes from VACV WR and IHD-J strains demonstrate contribution of cell-free virus to spreading. (A) Still micrographs of representative plaque phenotypes from VACV-IHD-J-E/L-GFP and VACV-WR-GFP in either gelled or liquid medium. (B) Example of VACV-WR-E/L-GFP live microscopy plaques (24 h pi) analyzed by Plaque2.0 software. Here superimposed on the micrograph from the GFP signal, green colored pixels designate foreground pixels detected by thresholding, colored line designates plaque borders and red spots highlight local intensity maxima. This procedure allows detection of adjacent plaques. (C) The relative plaque area normalized to the plaque area from gelled medium was plotted as a function of time. Note that VACV-IHD-J plaques occupy a larger area than VACV-WR at late stages of infection owing to cell-free EEV. Results represent averages from over 50 plaques for each condition from 8 technical replicas.

We investigated the differences in plaque phenotypes of VACV-IHDJ-E/L-GFP and VACV-WR-E/L-GFP infections. While the IHD-J strain yielded elongated comet-like plaques in liquid medium but not gelled medium, the WR strain gave spherical plaques under both conditions (Fig 3A). Similar to the results in Fig 1, the IHD-J comets contained spherical satellite plaques, unlike the WR strain, suggesting that they arise from mixed cell-free and cell-cell virus transmission events (Fig 3A and 3B).

Next, we tested the fidelity of image segmentation using the Hoechst nuclear stain as a benchmark. For this, we introduced synthetic images mimicking cell nuclei stained with Hoechst. The detection of seeded cells in synthetic images was linear with dose over a range of 1 log_10_ with a correlation factor R^2^ > 0.997 (Figs A-C in S2 Fig). The detection error at high seeding density (80’000 cells per well) was around 5% but at low cell density was less than 5% (Fig C in S2 Fig). In addition, we introduced the algorithm ‘Coarse and Fine Detection’ of plaques allows to enhance cell counting of even closely adjacent plaques. Thus, fluorescence imaging and Plaque2.0 image segmentation are sufficiently accurate for counting cells at low optical resolution.

Further, we analyzed the plaque area in liquid and gelled medium, respectively (Fig 3C). The relative plaque area of IHD-J infections (normalized to gelled medium) increased at about 30 h pi, and reached levels that were several fold higher than in gelled medium, while WR plaques were invariant (see S1, S2 and S3 Movies, and Figs A-C in S3 Fig, Figs A-D in S4 Fig). The time-lapse data further demonstrated that the comet tails of VACV-IHDJ-E/L-GFP were in part formed by the satellite plaques. This was not due to IHD-J virus replicating faster than the WR strain, as shown in time-lapse microscopy analyses of BSC40 cells infected with serial dilutions of either virus strain (Figs A-D in S4 Fig). The data thus show that the cell-free IHD-J viruses (mostly the EEV form) are more efficient long range spreaders than the cell-cell based WR viruses. These results extend the previously described mechanisms of enhanced the cell-cell transmission of VACV via actin tail formation [31, 51].

### Plaque2.0 scores plaques from cell-free spreading adenovirus

Classical infection assays measure indirect phenotypes without scoring viral proteins or nucleic acids, for example cell death in conventional plaque assays or in tissue culture infectious dose 50 (TCID_50_) assays. Alternatively, population-based fluorescence or luminescence measurements of transgene expression, immuno-fluorescence staining or counting of infected and uninfected cells are used. We developed Plaque2.0 to measure a combination of direct and indirect infection features based on multi-parametric datasets, including cell-based information, such as infection intensity of individual cells, and object-based information, such as viral plaque features.

**Fig 4:**
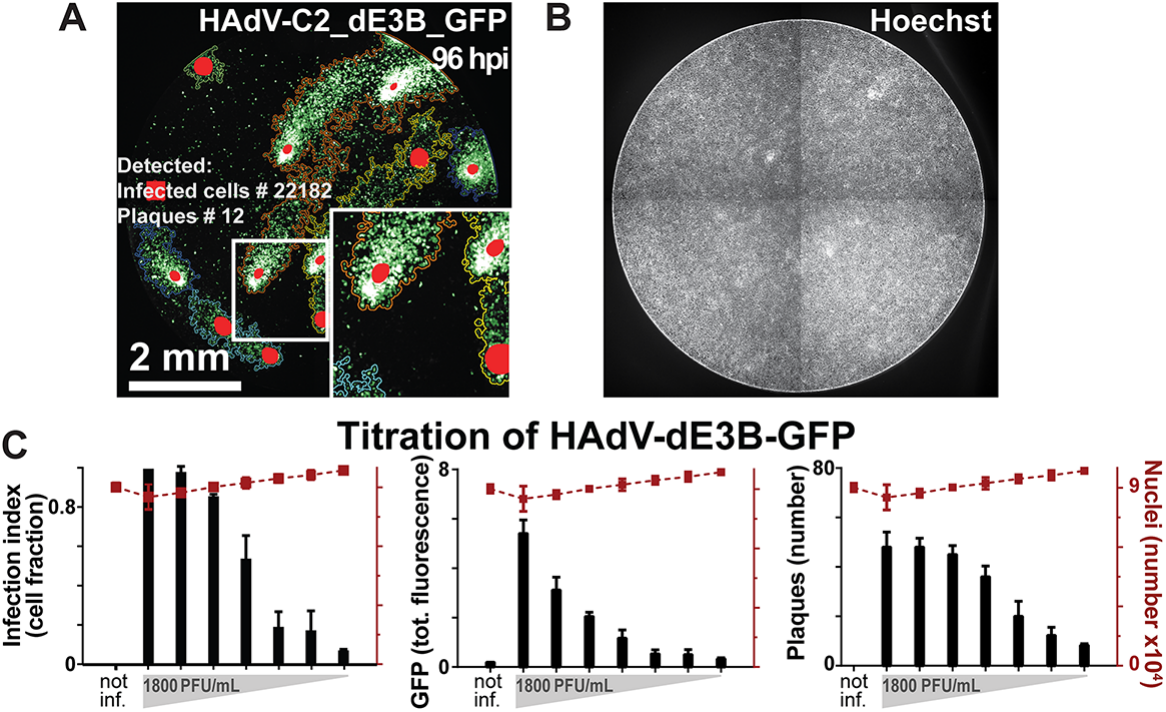
Analyses of HAdV infection by population or plaque phenotypes give similar results. (A) Plaques in 96 wells of A549 cells infected with HAdV-C2_dE3B_GFP were analyzed by thresholding GFP fluorescence with Plaque2.0 software 96 h pi. Colored lines designate plaque borders and red spots local intensity maxima. This enables to distinguish plaques in near proximity to each other. (B) Micrograph depicting the nuclei of the cells in (A) stained with Hoechst 33342. (C) Correlation of HAdV-C2_dE3B_GFP plaque formation measured by Plaque2.0, infection index defined as fraction of GFP-expressing cells per total cells, and total GFP intensity in a population assay of 2-fold serially diluted inoculum. Results are mean values from 3 replicas, and error bars represent the standard deviations of the respective means.

We infected monolayers of A549 human lung carcinoma cells with replication competent HAdV-C2_dE3B-GFP, and stained the nuclei with Hoechst 33342 (Fig 4A and 4B). GFP expression was an indication of infection, and Hoechst staining a surrogate for cell viability. We compared plaque numbers with the overall infection index, which was defined as the ratio of infected to uninfected cells, and the total infection signal measured as GFP intensity per well 72 h pi (Fig 4C). As expected, all the three readouts, i.e. the number of plaques, the infection index and the total fluorescence, were strictly dependent on the amount of virus inoculum. Remarkably, at very low amounts of inoculum, the total fluorescence was barely distinguishable from the background fluorescence, while the plaque number and infection index showed clear signal-noise separation. At very high amounts of inoculum (when all the cells were infected), the measurement of total fluorescence still increased with viral dose, while plaque number and infection index where saturated. This demonstrates complementarity of the readouts, and shows that Plaque2.0 expands the linear range of infection measurements.

### Plaque2.0 is suitable for high-throughput screening

**Fig 5:**
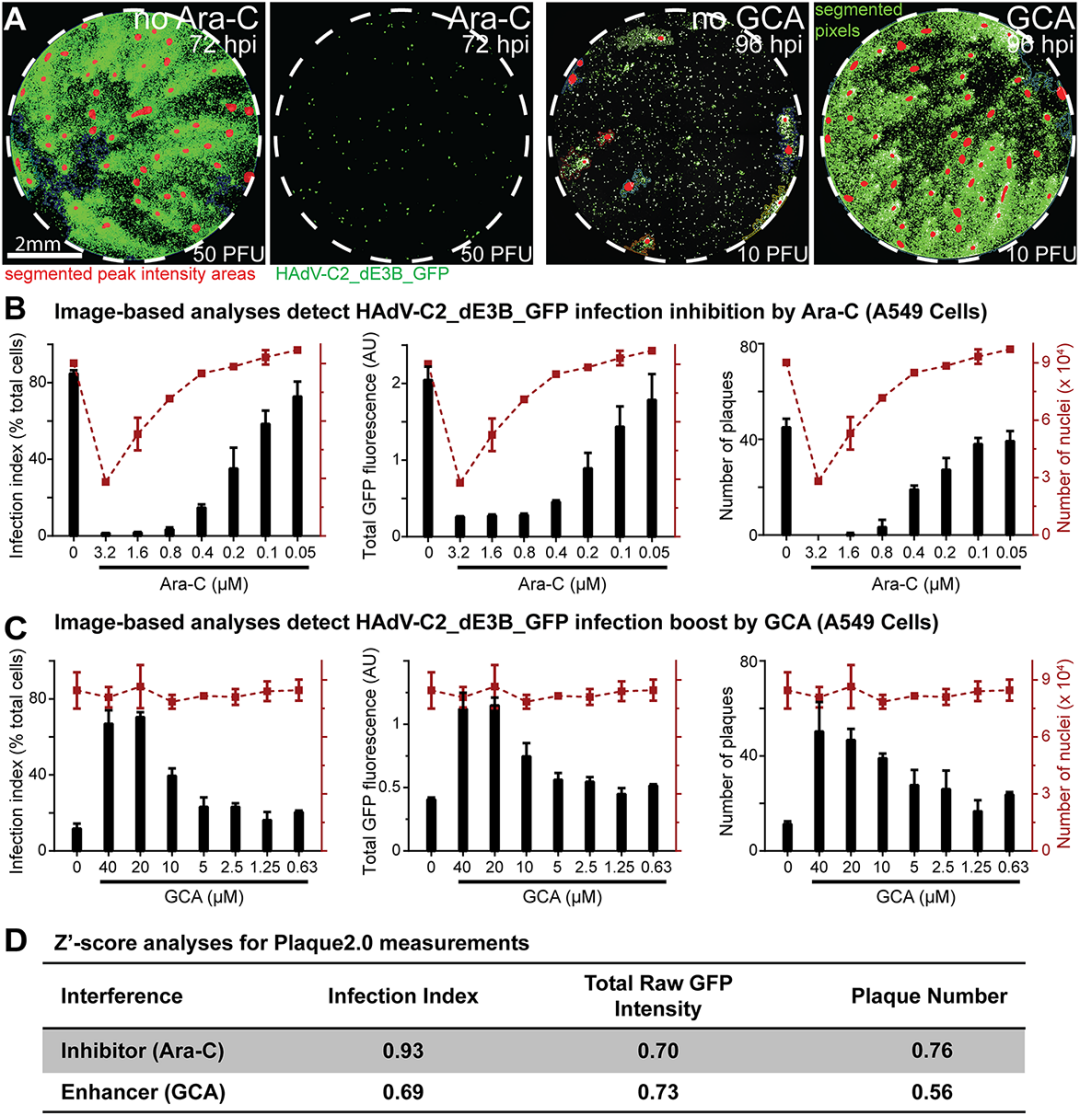
Plaque2.0 scores infection phenotypes in high throughput mode. (A) Effects of Ara-C (3.2 μM) and GCA (40 μM) on HAdV-C2_dE3B_GFP plaque formation in A549 cells 2 days pi with low MOI of 50 or 10 PFU per 96 well dish, respectively. Green signals designate infected cells (GFP), and red signals highlight local GFP maxima indicative of the center of the respective plaques. (B, C) Plaque2.0 scored HAdV-C2_dE3_GFP infection index, total GFP fluorescence and plaque numbers in A549 cells treated with Ara-C (B) or GCA (C). Red dotted lines represent the number of nuclei in the respective 96 wells. Results are represented as mean values from two technical replicas, and error bars represent the standard deviations of the respective means. (D) Z’-score analyses for Plaque2.0 assay of Ara-C (0.4 μM) or GCA (40 μM) tuned HAdV-C2_dE3B_GFP infections.

We next tested if Plaque2.0 was suitable for high-throughput screening assays (HTS). We scored infection with HAdV-C2_dEB_GFP using semi-automated liquid handling procedures on A549 cells treated with an inhibitor or an enhancer of DNA virus infection. For inhibition, we chose cytosine arabinoside (Ara-C), which inhibits DNA replication of HSV, VACV and HAdV [36, 52, 53], and for enhancement Golgicide A (GCA), which boosts early adenovirus gene expression [37]. Ara-C inhibited and GCA enhanced infection in a dose-dependent manner (Fig 5A). Infection index, total fluorescence, and number of plaques were strictly dependent on Ara-C and GCA doses (Fig 5B and 5C). At high concentrations of Ara-C, for example 4 μM Plaque2.0 scored cell toxicity but not at EC50 concentrations of 0.7 μM. Importantly, the liquid volumes in the 96 or 384-well formats were about 30-fold lower than in conventional plaque assay. Imaging and analyses of a 96 or 384-well plate took 15 and 15–30 min, respectively. In addition, the high sensitivity of fluorescence in scoring infected cells reduced the handling and infection times about two-fold compared to conventional plaque assay.

To assess the quality of the HTS assays we carried out Z’-score analyses taking into account readout values for positive and negative controls [45]. To avoid a cytotoxicity bias, nontoxic Ara-C concentrations were considered. At 0.4 μM AraC or 5 μM GCA, all infection readouts had Z’-scores between 0.5 and 1, and were separated from the controls by at least 3σ (Fig 5D). The data indicate that Plaque2.0 analyses deliver “excellent” quality according to the criteria described earlier [45]. We conclude that Plaque2.0 is well suited for HTS.

### In situ genotyping of human rhinoviruses in co-infections demonstrates independent infection spreading

**Fig 6:**
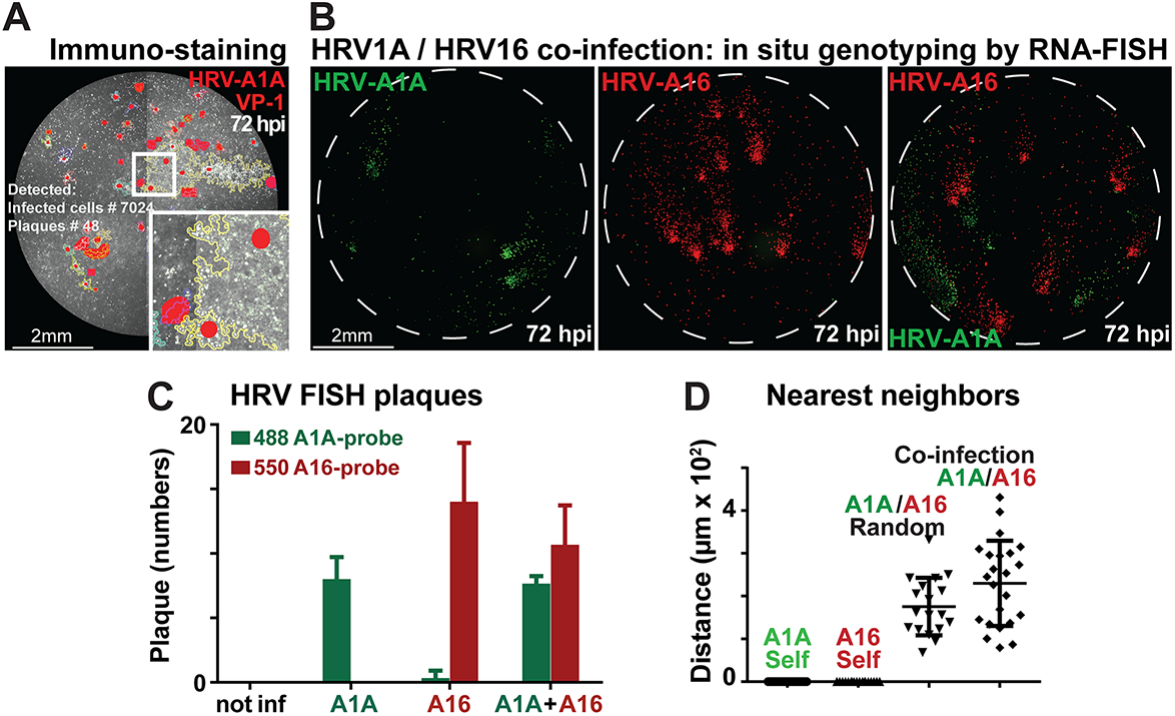
Fluorescence in situ hybridization scores HRV co-infections. (A) HRV-A1A infected HeLa cells were immunostained with anti-VP1 antibodies (white signal) 72 h pi, and processed by Plaque2.0 analyses. Yellow lines designate plaque borders and red signals local intensity maxima. (B) HRV-A1A and HRV-A16 co-infected HeLa cells were detected by RNA FISH probes stained at 488 nm (HRV-A1A, green signal) and 550 nm (HRV-A16, red signal) followed by Plaque2.0 analyses. Single infections with HRV-A1A and HRV-A16 are shown in the left and middle micrograph, respectively. (C) Bar graph of HRV-A1A and HRV-A16 plaque analyses by the Plaque2.0 software. Results from individual infections and co-infections are mean values from 3 replicas, and error bars represent the standard deviations of the respective means. (D) Nearest neighbor analyses of plaque centers from HRV-A1A and HRV-A16 infections. The nearest neighbor distances between HRV-A1A and HRV-A16 plaque centroids were not different in single infections (random) or co-infections (i.e. random). Note that self co-localization control was close to zero, as expected. Results are mean values from 3 replicas containing at least 18 plaques per condition, and error bars represent the standard deviations of the respective means.

So far, we have shown that Plaque2.0 scores infection phenotypes from GFP-expressing reporter viruses. We next analyzed plaques from wild type viruses by immuno-staining newly synthesized viral protein 1 (VP1) of human rhinovirus A1A (HRV-A1A) 72 h pi (Fig 6A). In humans, HRVs cause the common cold, frequently as a result of co-infections by different HRV types [54]. We analyzed if Plaque2.0 scores plaques in co-infections of HeLa cells by HRV-A1A and HRV-A16 serotypes at very low MOI yielding about 10 plaques from each HRV type, and visualized the infected cells by genotype specific RNA FISH (Fig 6B and 6C). Similar to the GFP-expressing VACV and HAdV, we scored several hundred VP1-positive cells per plaque. To determine the extent of plaque overlap between the two genotypes we measured the distance between a plaque of one genotype and its nearest neighbor plaque of another genotype using location readout from Plaque2.0 (Fig 6D). We introduced two internal controls, one mimicking perfect co-localization (complete overlap of the signals), and the other one perfect random co-localization. In the first case, co-localization of the plaques of single genotypes to themselves was measured (“HRV-A1A Self” and “HRV-A16 Self”). As expected the nearest neighbor distances were equal to zero. In the second case, nearest neighbor distances between plaques of two genotypes obtained from separate wells of single virus infected cells were measured. This case is designated “Random”. We compared the results of these controls to the co-localization of plaques from mixed infections. Results showed that Plaque2.0 unequivocally detected HRV type-specific plaques with an average distance of about 200 μm (Fig 6D). The data indicate that spreading infections with HRV-A1A or HRV-A16 do not overlap. This implies that a single infectious event can result in a plaque without the contribution of other infectious agents in the inoculum.

### Plaque2.0 detects colonies of cancer cells in co-culture conditions

**Fig 7:**
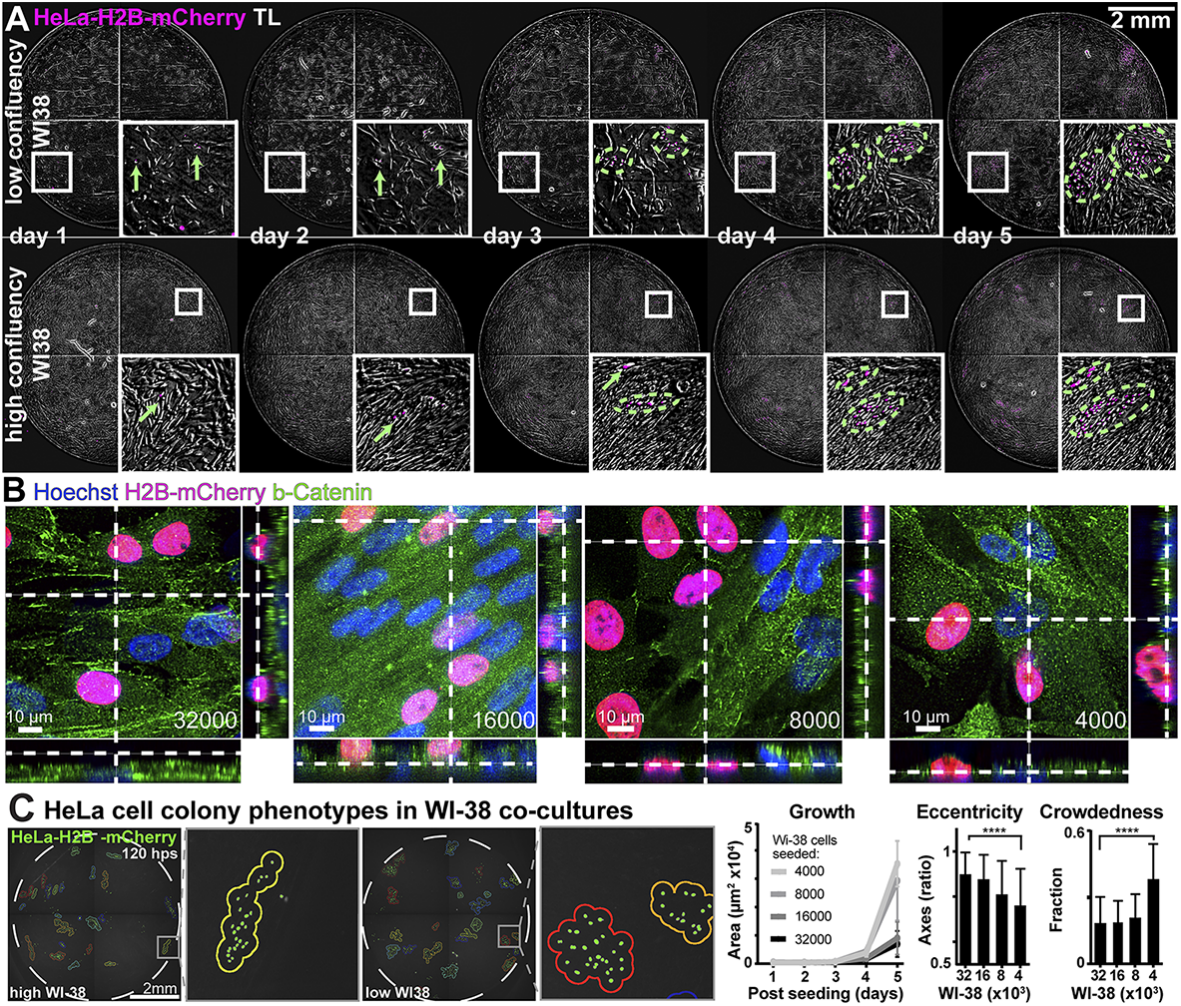
Cell density dependent features of clonal cancer cell growth in co-cultures with normal cells. (A) Time-lapse analyses of HeLa-H2B-mCherry cell colony formation on WI-38 fibroblasts at sub-confluent (4000 WI-38 cells per well) or confluent density (32000 cells per well). Colony outlines are indicated by dashed green lines. (B) Confocal fluorescence microscopy of HeLa-H2B-mCherry cocultured with 32000, 16000, 8000 or 4000 WI-38 cells (cell numbers determined at seeding time). The samples were fixed 5 days post HeLa cell seeding. Cells were stained with Hoechst nuclear dye (blue) and immuno-stained for ß-catenin (green). Z-sections across the cultures along the dotted lines are shown on the right side and the bottom of the images. (C) Colony phenotypes of HeLa-H2B-mCherry cells co-cultured with WI-38 fibroblasts upon live-imaging and analysis with Plaque2.0. The area of the colonies is a measure of growth cell growth, the eccentricity of the elliptic colony shapes, defined as the ratio of the distance of the elliptic foci to the major axis, serves as an indicator of the cell environment, and the crowdedness fraction is an indicator of the HeLa cell colony density. Mean values from 3 technical replicas containing at least 60 individual colonies per condition are shown, and error bars represent the standard deviations of the respective means. Statistical significance was determined by the Komogorov-Smirnov nonparametric test.

Clonal phenotypes are typical features for viral plaques, and also describe propagation of cancer cells among normal cells. We thus tested if Plaque2.0 detected cancer cell colonies. HeLa cells stably expressing mCherry-tagged histone H2B (HeLa-H2B) were seeded at low concentration among non-labeled normal diploid human lung WI-38 fibroblasts grown to different confluency. Live fluorescence and transmission light microscopy revealed that the shape of the HeLa-H2B colonies was significantly different depending on the confluency of the WI-38 cells (Fig 7A). In case of low WI-38 confluency HeLa-H2B cells were growing in the same Z-plane as WI-38, whereas at high confluency they grew on top of WI-38 cells, as indicated by z-stack confocal microscopy analyses (Fig 7B). We measured the eccentricity of the HeLa-H2B colonies at different WI-38 densities. Eccentricity is defined as the ratio of the distance between the two foci of the ellipse to the length of the major axis, equaling zero for a circle. At low WI-38 density, the HeLa-H2B colonies occupied a larger area and had a lower eccentricity than at high WI-38 density (Fig 7C). At high WI-38 confluence, the shape of HeLa colonies was highly eccentric, unlike at low WI-38 confluence. Plaque2.0 also determined the crowdedness of the colonies, that is the fraction of HeLa-H2B cells within a colony. Interestingly, the crowdedness was largely independent of the WI-38 confluency, although it was significantly higher at the lowest confluency of 4000 WI-38 cells per 96 well compared to 8000. Taken together, the analyses showed that Plaque2.0 is suitable to characterize clonal growth properties of cancer cells. The data demonstrated that HeLa cells preferentially adhered to sites on the dish that are free of WI-38 cells. Particularly highly confluent WI-38 cells slowed down the growth of HeLa colonies.

## Discussion

Clonal cell properties are an important feature of biological matter, but they have not been systematically analyzed. One reason is that available image analyses methods largely deal with subcellular structures rather than features of cell populations [55–57]. Here we provide a customized open-source software solution for analyses of clonal cell features, Plaque2.0. Plaque2.0 is distinct from existing software, such as ‘CellProfiler’ or ‘Icy’ [56, 58], as it provides means to quantify features of cell populations in low magnification images. It reliably and rapidly analyzes images of large size, such as 4320x4320 pixels, where analyses by ‘CellProfiler’ are too slow for scoring multicellular objects. Plaque2.0 is scalable and delivers multiple readouts with high precision and a wide dynamic range. It has been optimized for detecting microscopic fluorescent plaques, cell colonies or cell nuclei in confluent or sub-confluent monolayers, and provides multi-parametric datasets, including object-based information of cell clones, and information from thousands of single cells.

The imaging equipment that we used in Plaque2.0 operates through a 4x midrange magnification lens, and a high resolution sCMOS (scientific Complementary Metal Oxide Silicon) camera. This set-up can be used for immunofluorescence analyses, FISH and also live cell experiments. It enables ultra-fast automated fluorescence imaging of micro-plaque phenotypes. Importantly, Plaque2.0 allows for tracking of infection progression or clonal cancer cell propagation from micro-scale at the cellular level to macro-scale at the plaque level in live mode.

For the study here, we used three different infection assays, transgenic fluorophore expression, immunofluorescence and RNA-FISH. In all cases, and for all the viruses tested, the fluorescent plaques had a single bright origin and a relatively dim periphery. These features allowed the unequivocal identification of the plaque centroid, and enhanced the identification of plaques even at high levels of infection. The use of fluorescence imaging combined with Plaque2.0 analyses gives another important advantage over traditional plaque assay, namely the distinction of infections that lead to viral spreading from those that are restricted to a single cells without spreading. This feature was originally described for HAdV [13], and can now be systematically analyzed for any other virus.

The shape of a plaque is the key diagnostic feature for the mode of virus transmission between cells. Plaque2.0 scored spherical plaques from non-lytic infections, and comet-shaped plaques from lytic infections by enveloped and non-enveloped RNA and DNA viruses. We used five replication-competent viruses from three different families, that is, two VACV strains (WR and IHD-J), one adenovirus (HAdV-C2_dE3B_GFP), and two rhinoviruses (HRV-A1A, and HRV-A16), as well as herpes virus (HSV-1-GFP, data not shown). These viruses spread from cell-to-cell through lytic or non-lytic routes [9, 13, 15, 17, 59, 60]. For example, we found here that the comet-shaped plaques of VACV-IHD-J are formed by cell-free virus, which spreads over the cell monolayer by convective currents in the medium, similar to adenovirus [13]. These findings extend an earlier study which showed that the rate of infection and the spreading efficiency of cell-associated VACV is higher than that of cell-free VACV [31]. Plaque2.0 documented a dual mode of VACV transmission by demonstrating that VACV-IHD-J plaques were comet-shaped, and contained spherical satellite plaques. The infected cell that gave raise to the spheroid plaque was initially derived from an extracellular virus that was released from the donor cell of the comet-shaped plaque.

Plaque2.0 is scalable at high precision, and its applications go beyond characterizing viral infections. For example, we showed that Plaque2.0 allows for analyses of cellular crowding. By characterizing clonal growth properties of HeLa cancer cells, we provided evidence that cancer cells spread clonally in cell cultures. This suggests that Plaque2.0 is suitable to analyze general cell biological phenomena, and can, for example, be applied to study metastatic cancer cell spreading in an organism. Further to this, Plaque2.0 may be useful to track cell lineages from precursor cells, for example in cancer metastases, stem cell research, or developmental and regenerative biology [61, 62].

In conclusion, Plaque2.0 is a fast, efficient and accurate method to analyze clonal cell features. Assays compatible for Plaque2.0 analyses consume less reagents than conventional plaque assay. Plaque2.0 is well suited for high-content screening, data acquisition and analysis. This allows assessments of systems properties and biological variability. In addition, Plaque2.0 delivers quantitative dynamic information in time-lapse mode, which can be used to reveal new features of viral spreading. For example, it analyzes cell biological and pathological features of co-infections by closely related viruses, or unrelated viruses, and it bears the potential to be adapted to complex biological samples, such as organoids or tissues.

## Supporting Information

S1 Fig Quantification of VACV present in the cell-free medium. Supernatant from wells infected with either VACV-IHD-J-E/L-GFP or VACV-WR-E/L-GFP was diluted 5-fold with cell culture medium, and transferred to fresh wells using classical plaque assay protocol (**Fig A**). Scanned pictures of wells from a time course experiment 3 to 21 h pi are shown. Time-resolved analysis of the amount of extracellular virus (EEV) produced in the transfer experiment (**Fig B**). Results are expressed as the PFU ratio of input to output virus, and are representative of three experiments with similar results. Titration of VACV-IHD-J-E/L-GFP from the supernatant of infected cells harvested 18 h pi (**Fig C**). Results are representative of three experiments with similar results.
(TIF)

S2 Fig Benchmarking the reliability of fluorescent cell detection. The segmentation of nuclei stained with Hoechst was benchmarked by comparison to synthetic images with a known number of cells (**Fig A**). Micrographs of A549 cells stained with Hoechst were obtained with ImageXpress Micro XL using 4x objective (left). Synthetic micrographs of nuclei (based real micrographs from ImageXpress Micro XL 4x objective) were placed in a pattern mimicking cells in a well of a 96 well. Correlation between seeded and detected synthetic nuclei, analyzed by Plaque2.0 software (**Fig B**). Plot of the segmentation error depending on the number of synthetic seeded nuclei (**Fig C**).
(TIF)

S3 Fig Still analysis of time-lapse microscopy of VACV IHD-J and WR strains expressing GFP in liquid or semi-solid medium. Merge of transmission light, propidium iodide (PI) indicating dead cells, and GFP signal indicating infection 50 h pi (**Fig A**). Color-coded GFP intensity representation 50 h pi (**Fig B**). Color-coded GFP intensity representation of time points 22 to 47 h pi depicting representative differences in IHD-J plaque phenotypes (**Fig C**).
(TIF)

S4 Fig Time-lapse microscopy of infection with VACV IHD-J and WR strains. Color-coded GFP intensity in still images from infections at different MOI 12.3 h pi (**Fig A**). The montage of representative micrographs from 96-well micro-titer plates reveals that the GFP intensity depends on the amount of input virus. Time resolved analyses similar as in Figure A (**Fig B**). The data represent transgene expression over time from cells infected with highest amount of either VACV-WR-E/L-GFP or VACV-IHD-J-E/L-GFP. VACV-WR-E/L-GFP or VACV-IHD-J-E/L-GFP dose-dependent GFP intensity and fraction of infected cells at 12.3 h pi (**Fig C and Fig D**).
(TIF)

S1 Movie Time-lapse microscopy of VACV plaque formation suggesting that cell-free virus contributes to spreading. Merged movie of transmission light, propidium iodide (PI) and GFP signal from cells infected with VACV-WR-E/L-GFP or VACV-IHD-J-E/L-GFP.
(MOV)

S2 Movie Time-lapse microscopy of VACV titration. Time-lapse imaging of cells infected with VACV-WR-E/L-GFP or VACV-IHD-J-E/L-GFP. Each square represents a well with a respective virus concentration from a serial dilution. GFP intensity was color-coded.
(MOV)

S3 Movie Time-lapse microscopy of VACV titration. Time-lapse imaging of cells infected with VACV-WR-E/L-GFP or VACV-IHD-J-E/L-GFP. Each square represents a well with a respective virus concentration from a serial dilution. GFP intensity was color-coded.
(MOV)
