# Supplementary figures and images for: Plaque2.0—A High-Throughput Analysis Framework to Score Virus-Cell Transmission and Clonal Cell Expansion
Source: PLoS One. 2015 Sep 28;10(9):e0138760. doi: 10.1371/journal.pone.0138760 (PMC4587671; doi:10.1371/journal.pone.0138760)

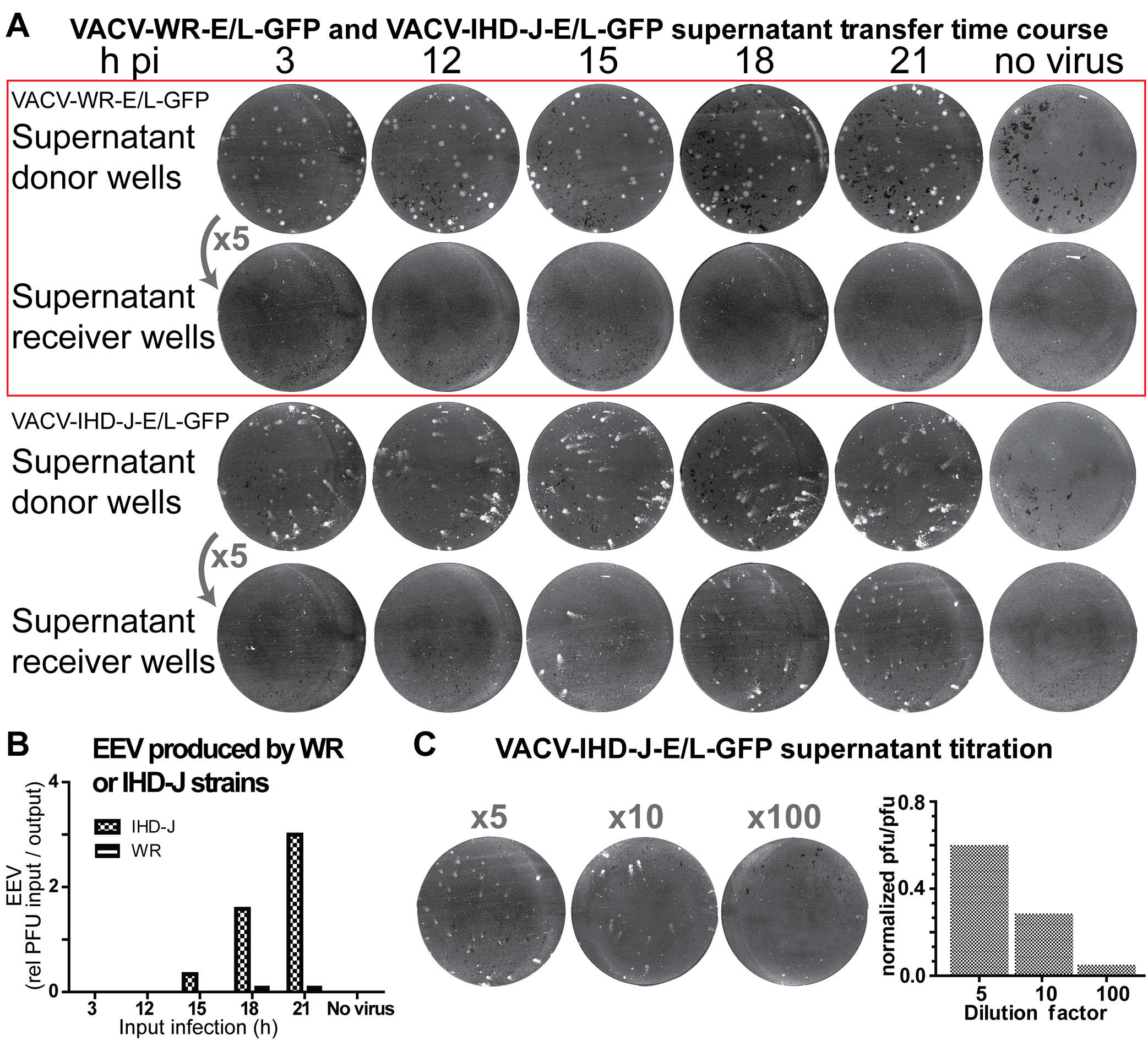

Supplement: S1 Fig — Supernatant from wells infected with either VACV-IHD-J-E/L-GFP or VACV-WR-E/L-GFP was diluted 5-fold with cell culture medium, and transferred to fresh wells using classical plaque assay protocol (Fig A). Scanned pictures of wells from a time course experiment 3 to 21 h pi are shown. Time-resolved analysis of the amount of extracellular virus (EEV) produced in the transfer experiment (Fig B). Results are expressed as the PFU ratio of input to output virus, and are representative of three experiments with similar results. Titration of VACV-IHD-J-E/L-GFP from the supernatant of infected cells harvested 18 h pi (Fig C). Results are representative of three experiments with similar results. (TIF) [file pone.0138760.s001.tif]

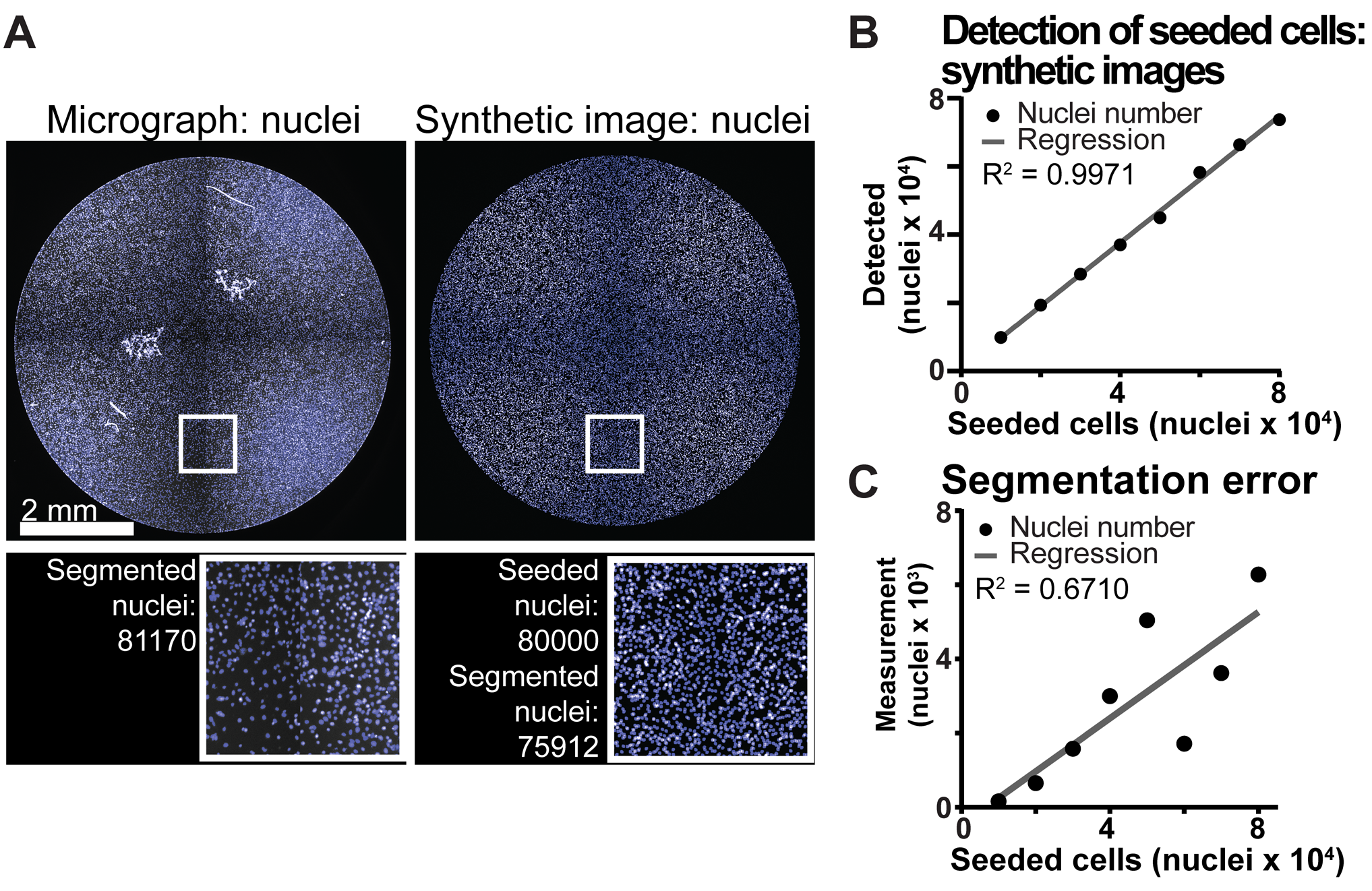

Supplement: S2 Fig — The segmentation of nuclei stained with Hoechst was benchmarked by comparison to synthetic images with a known number of cells (Fig A). Micrographs of A549 cells stained with Hoechst were obtained with ImageXpress Micro XL using 4x objective (left). Synthetic micrographs of nuclei (based real micrographs from ImageXpress Micro XL 4x objective) were placed in a pattern mimicking cells in a well of a 96 well. Correlation between seeded and detected synthetic nuclei, analyzed by Plaque2.0 software (Fig B). Plot of the segmentation error depending on the number of synthetic seeded nuclei (Fig C). (TIF) [file pone.0138760.s002.tif]

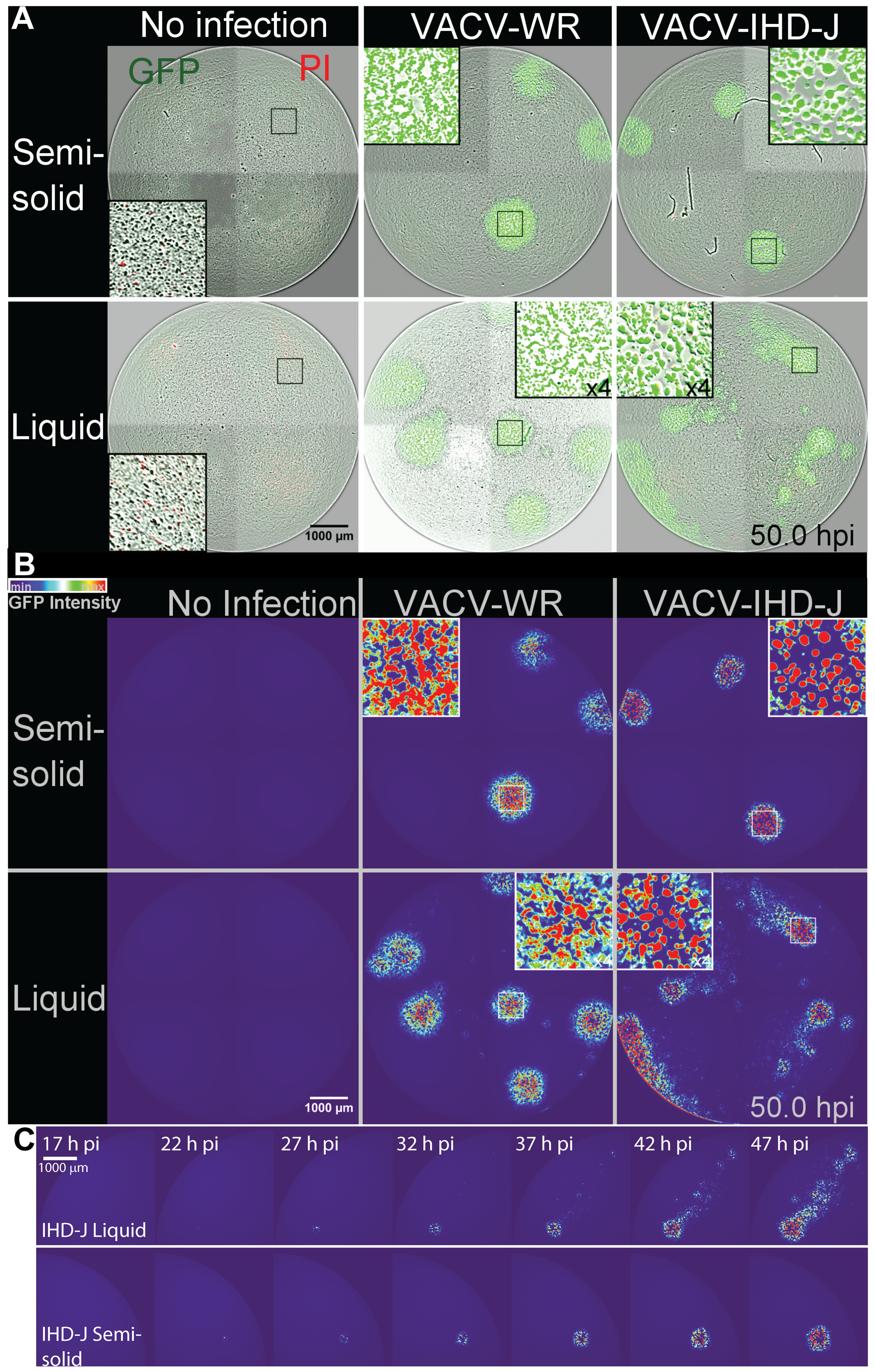

Supplement: S3 Fig — Merge of transmission light, propidium iodide (PI) indicating dead cells, and GFP signal indicating infection 50 h pi (Fig A). Color-coded GFP intensity representation 50 h pi (Fig B). Color-coded GFP intensity representation of time points 22 to 47 h pi depicting representative differences in IHD-J plaque phenotypes (Fig C). (TIF) [file pone.0138760.s003.tif]

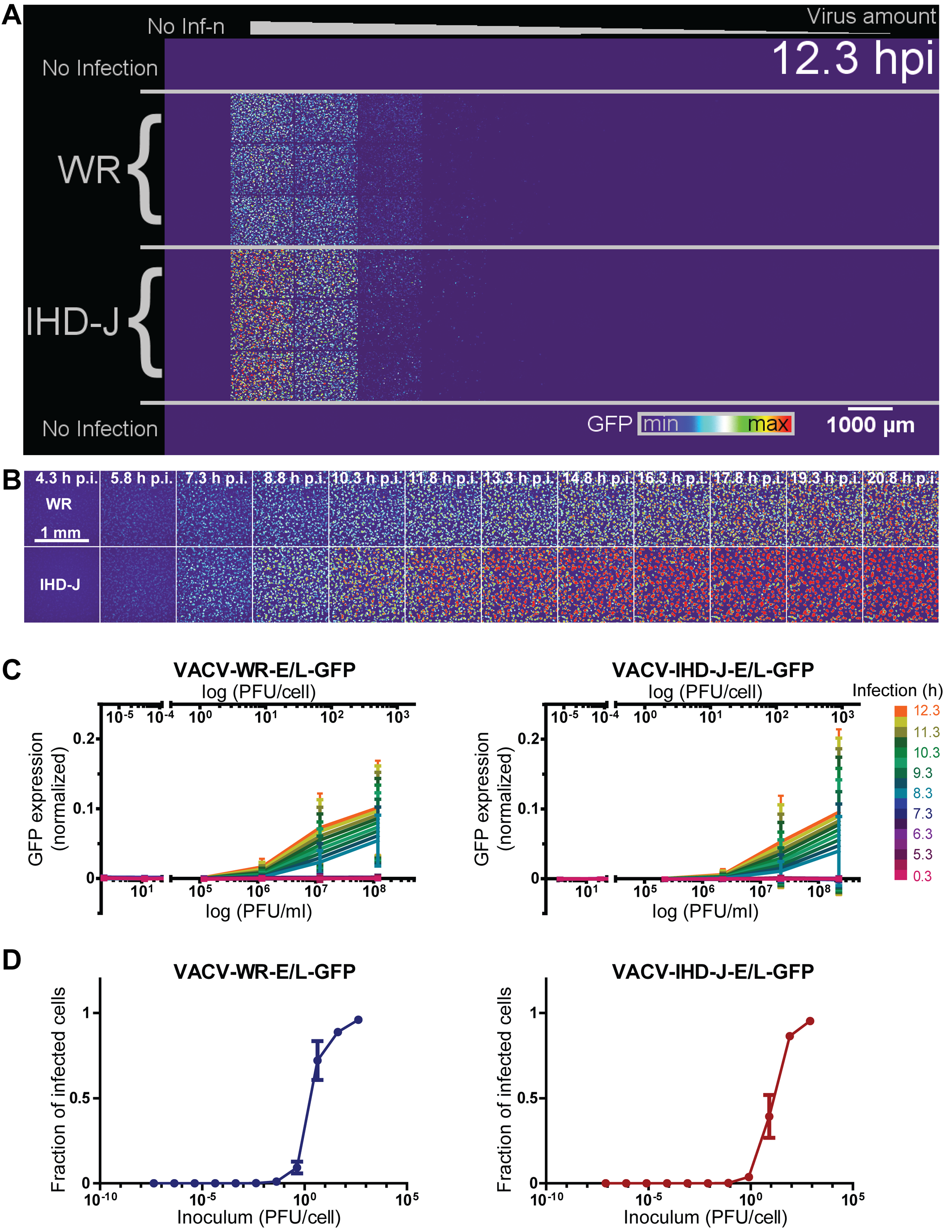

Supplement: S4 Fig — Color-coded GFP intensity in still images from infections at different MOI 12.3 h pi (Fig A). The montage of representative micrographs from 96-well micro-titer plates reveals that the GFP intensity depends on the amount of input virus. Time resolved analyses similar as in Figure A (Fig B). The data represent transgene expression over time from cells infected with highest amount of either VACV-WR-E/L-GFP or VACV-IHD-J-E/L-GFP. VACV-WR-E/L-GFP or VACV-IHD-J-E/L-GFP dose-dependent GFP intensity and fraction of infected cells at 12.3 h pi (Fig C and Fig D). (TIF) [file pone.0138760.s004.tif]
